# C57BL/6N Albino/Agouti Mutant Mice as Embryo Donors for Efficient Germline Transmission of C57BL/6 ES Cells

**DOI:** 10.1371/journal.pone.0090570

**Published:** 2014-03-05

**Authors:** Branko Zevnik, Nathalie C. Uyttersprot, Ana V. Perez, Gerald W. M. Bothe, Heidrun Kern, Gunther Kauselmann

**Affiliations:** 1 Cologne Excellence Cluster on Cellular Stress Responses in Aging-associated Diseases (CECAD), University of Cologne, Cologne, Germany; 2 TaconicArtemis GmbH, Cologne, Germany; 3 Taconic, Hudson, New York, United States of America; Wellcome Trust Centre for Stem Cell Research, United Kingdom

## Abstract

We generated C57BL/6NTac mice carrying a tyrosinase loss-of function mutation and a reversion of the nonagouti locus to agouti. This strain has a high superovulation response, allows visual detection of chimeric coat color contribution of C57BL/6 ES-cells and provides a simplified breeding format that generates black G1 offspring of pure inbred C57BL/6 background in one step, providing the ideal host for genetically manipulated C57BL/6 ES cells.

## Introduction

Introduction of gene-targeted mouse embryonic stem (ES) cells into blastocysts host embryos is frequently used for the production of chimeric animals that when bred with the desired inbred strain would produce offspring carrying the desired genetic alteration. The extent of integration of the ES cells into the developing embryo proper including its germline is visually assessed in chimeras and G1 offspring by coat color differences between ES cells and the host embryo donor.

C57BL/6 is the most widely accepted murine inbred strain used for the creation of genetically engineered animal models. C57BL/6 mice display good breeding and reproductive characteristics, and are well defined. Consequently, members of the International Knockout Mouse Consortium (IKMC), aimed to provide mutated mouse ES cells in every protein-coding gene, and decided on the use of C57BL/6N ES cells [1].

Inbred strains, such as the naturally tyrosinase deficient albino BALB/c provide a suitable environment for the colonization with C57BL/6 derived ES cells [2]–[7] and allow at the same time simple recognition of ES cell contribution to chimeras by coat color. However, there are reported limitations on the use of BALB/c as an embryo donor, such as substrain-dependent poor response to superovulation [8], semifertility [9] and a delayed and unequal embryonic development of suitable blastocyts produced for microinjection [3], [7]. Other alternative embryo host/B6 ES cell combinations have been investigated, for example C3HxBALB/c [10], C57BL/6J [11] or tyrosinase-deficient albino C57BL/6 mouse strains isolated as spontaneous mutations in C57BL/6-*Tyr^c-Brd^* [12], B6(Cg)-*Tyr^c-2J^* [3] and more recently C57BL/6N-*Tyr^cWTSI^* [13]. However, besides limited availability of some of these strains, their major drawbacks are i) the inability to detect germline transmission of the mutated C57BL/6 ES cell genome by simple coat color assessment and ii) the difficulty in recovering a pure inbred C57BL/6 background from germline transmitted mice, since an additional time-consuming breeding step is needed to maintain the mutation on a pure C57BL/6 background without coat color mutations.

An alternative approach was used by Pettit et al to generate the heterozygous restoration of the agouti locus in C57BL/6NTac ES cells [12]. However in this reference, visual recognition of germline transmission is only possible for 50% of the G1 offspring when breeding to C57BL/6 mating partners. In addition, G1 germline offspring carry the mutated agouti allele including the residual Flp recombinase target site (Frt).

We sought to circumvent these drawbacks by using gene targeting to i) introduce the tyrosinase (albino)-mutation and ii) restore the agouti locus in a high superovulating inbred strain such as C57BL/6NTac. The presence of both mutations in a homozygous fashion allows for the visualization of black B6 or agouti B6 ES-derived offspring by coat color to recover pure C57BL/6 inbred mice from germline G1 crosses without any coat color mutation being transmitted to germline G1 offspring.

## Results and Discussion

**Figure 1:**
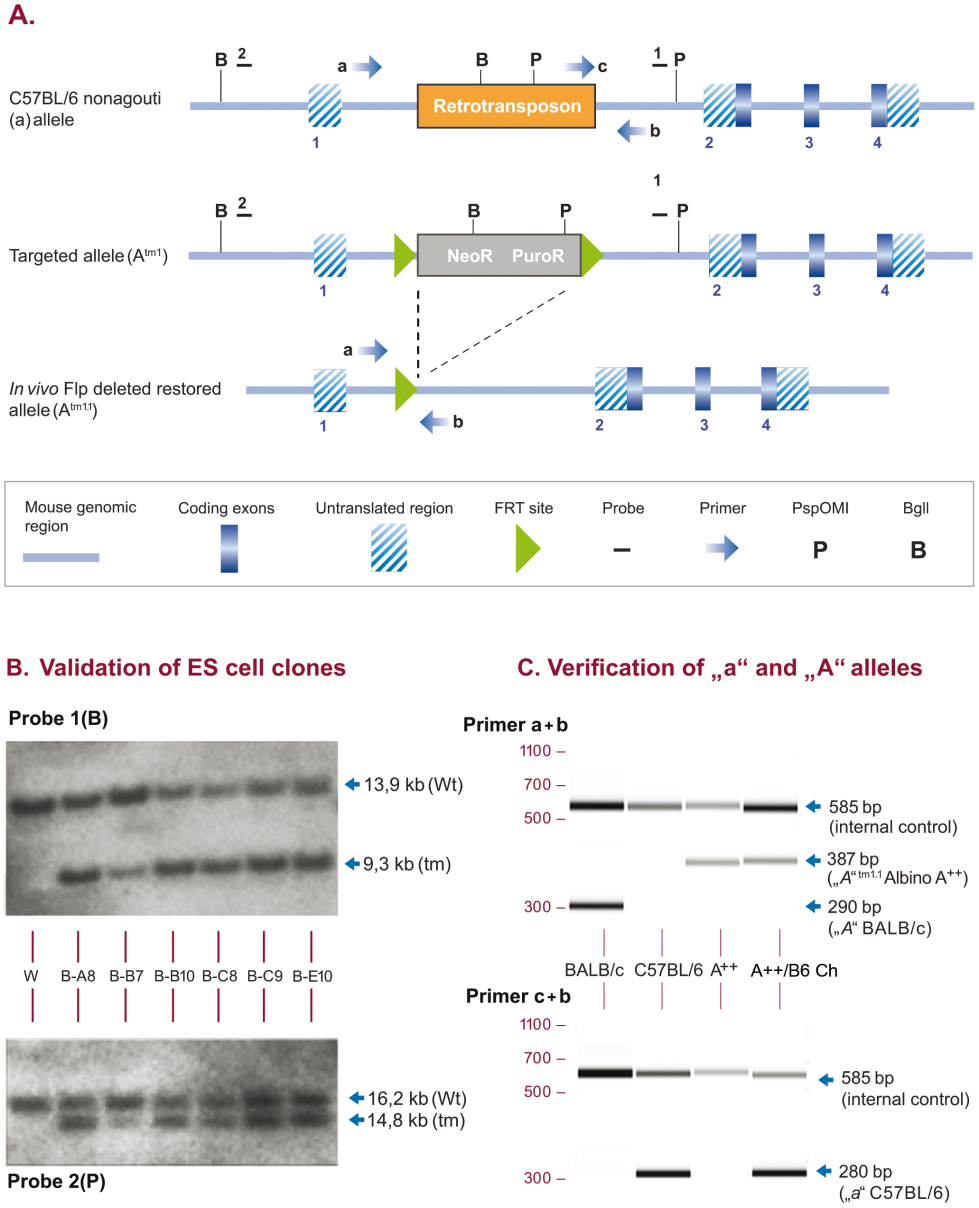
Restoration of the agouti locus in C57BL/6NTac ES cells and mice. (A) Gene targeting strategy. The Neomycin (NeoR)/Puromycin (PuroR) selection marker is displayed as a grey box (neoR and puroR). Numbered dashes indicate external probes used for Southern Blot analysis of ES cell clones, lettered arrows indicate oligos used for genotyping of mice. Deletion of the FRT flanked selection was obtained in vivo simultaneously with germline transmission. (**B**) Southern Blot validation with genomic DNA isolated from 6 ES cell clones and wildtype C57BL/6 genomic DNA as a control (W) using external probe 1 in combination with BglI restriction digest (upper panel, Probe 1, B) leading to a wildtype allele of 13,9 kb (Wt) and a targeted allele of 9,3 kb (tm) and confirmatory Southern Blot validation with external probe 2 in combination with PspOMI restriction digest (lower panel, Probe 2, P), leading to a wildtype allele of 16,2 kb (Wt) and a targeted allele of 14,8 kb (tm). (**C**) PCR verification. Clone B-B10 was selected for chimera generation and germline transmission. Primer combinations a + b (upper panel) resulted in amplification of a 290 bp wildtype *A* allele in BALB/c control mice (lane 1, BALB/c) and a 387 bp restored *A^tm1.1^* allele in homozygous Albino A++ mice (lane 3,A++) and in chimeras generated with C57BL/6 ES cells injected in homozyogus A++ host embryos (lane 4, A++/B6 Ch). Primer combinations c + b (lower panel) amplified a 280 bp C57BL/6 wildtype *a* allele in C57BL/6 control mice (lane 2, C57BL/6) and in A++/B6 Ch (lane 4, A++/B6 Ch). Note: PCR amplicons are of different size for the BALB/c *A* and the A++ restored *A^tm1.1^* alleles. Also, in contrast to homozygous A++ mice, A++/B6 Ch amplify both, the ES cell derived *a* and the A++ derived *A^tm1.1^* alleles.

Agouti is regarded to be the true wild-type coat color of mice. The dominant agouti (A) locus, situated on mouse chromosome 2 primarily affects the relative amount and the distribution of yellow and black pigments in hairs of the coat across the dorsal and ventral body surfaces [14]. The black coat color of C57BL/6 mice is caused by a 14.7 kb retrotransposon insertion into intron 1 of the nonagouti allele (NCBI gene ID: 50518) that selectively inactivates the expression of different isoforms of agouti transcripts [15]. We designed a gene targeting strategy to restore the dominant agouti allele (A) in C57BL/6NTac ES cells by deletion of the 14.7 kb retrotransposon sequence in intron 1 (Fig 1A). The correct introduction of the modification was validated by Southern Blot analysis (Fig 1B). Germline transmission and simultaneous excision of the Frt flanked selection marker cassette was achieved by crossing to Flp-deleter (C57BL/6NTac-Tg(CAG-Flpe)2Arte) mice, the correct genotype of C57BL/6NTac-*A^tm1.1Arte^* mice was verified by PCR (Fig. 1C) and visible by the change of the coat color pigmentation from black to agouti.

**Figure 2:**
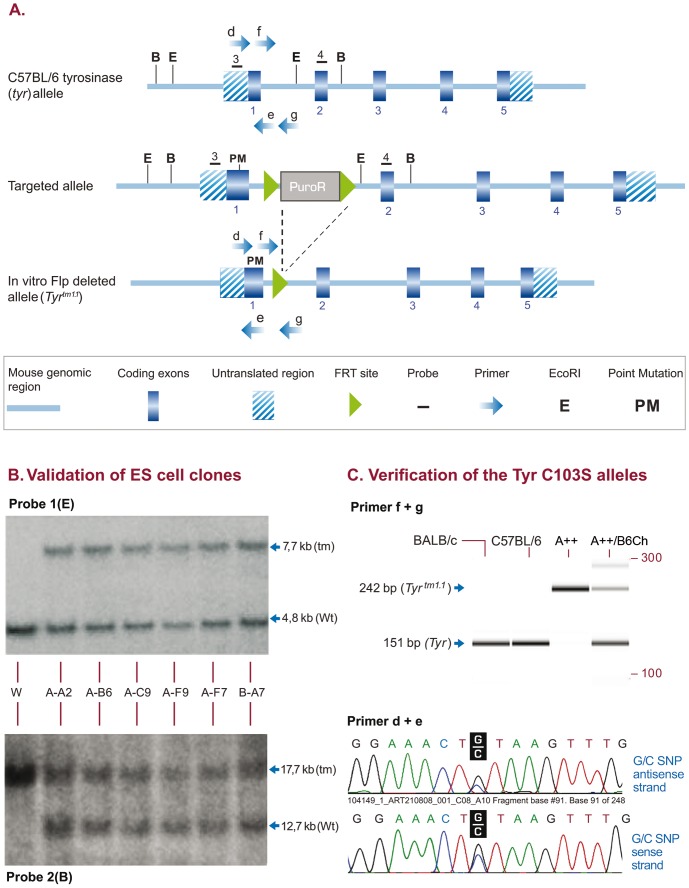
Inactivation of the tyrosinase locus in C57BL/6NTac ES cells and mice. (A) Gene targeting scheme for the tyrosinase locus. A point mutation was introduced in exon 1 (C103S) of the tyrosinase allele. The Puromycin selection marker (PuroR) is shown as a grey box. Numbered dashes indicate external probes used for Southern Blot analysis of ES cell clones, lettered arrows indicate oligos used for genotyping of mice. Deletion of the F3 flanked selection marker was achieved in vitro in targeted ES cell clone A-B6. (**B**) Southern Blot validation with genomic DNA isolated from 6 ES cell clones and wildtype C57BL/6 genomic DNA as a control (W) using external probe 1 in combination with EcoRI restriction digest (upper panel, Probe 1, E) leading to a wildtype allele of 4,8 kb (Wt) and a targeted allele of 7,7 kb (tm) and confirmatory Southern Blot validation with external probe 2 in combination with BglI restriction digest (lower panel, Probe 2, B), leading to a wildtype allele of 12,7 kb (Wt) and a targeted allele of 17,7 kb (tm). (**C**) PCR verification. Clone A-B6 was selected for chimera generation and germline transmission. Primer combinations f + g (upper panel) applied on genomic DNA displayed a 151 bp wildtype *Tyr* allele in BALB/c control mice (lane 1, Balb/c), C57BL/6 control mice (lane 2, C57BL/6) and in chimeras generated with C57BL/6 ES cells injected in homozyogus A++ host embryos (lane 4, A++/B6 Ch). In addition the modified *Tyr^tm1^*Arte allele was detected in homozygous Albino A++ mice (lane 3, A++) and in chimeras generated with C57BL/6 ES cells injected in homozyogus A++ host embryos (lane 4, A++/B6 Ch). Lower panel: the introduced point mutation was verified by sequencing both strands of the PCR fragment amplified with primers d + e.

The *Tyr* gene is located on chromosome 7 of the mouse genome [16]. The locus encodes for an enzyme involved in the synthesis of the hair follicle pigment in melanocytes. Defects in the tyrosinase protein have an epistatic character over all other coat color genotypes. Albinism is characterized by decreased or complete lack of pigmentation in melanocytes. Albinism in the BALB/c strain is caused by a G to C nucleotide transition at position 308 of the tyrosinase gene resulting in an amino acid substitution (Cys by Ser) at position 103 (NCBI gene ID: 22173) and the inactivation of the tyrosinase protein [17]. We performed an analogous replacement in C57BL/6NTac ES cells by applying a constitutive knock-in targeting strategy (Fig. 2A). The point mutation was validated by Southern blotting (Fig. 2B). The selection marker was removed *in vitro* by Flp recombinase. Germline transmission was achieved by crossing to C57BL/6NTac mice and confirmed by PCR and subsequent sequencing of the PCR fragment (Fig. 2C). Heterozygous mice were bred to homozygosity (C57BL/6NTac-*Tyr^tm1Arte^*) and albino mice were produced, showing lack of pigmentation in fur and eyes.

**Figure 3:**
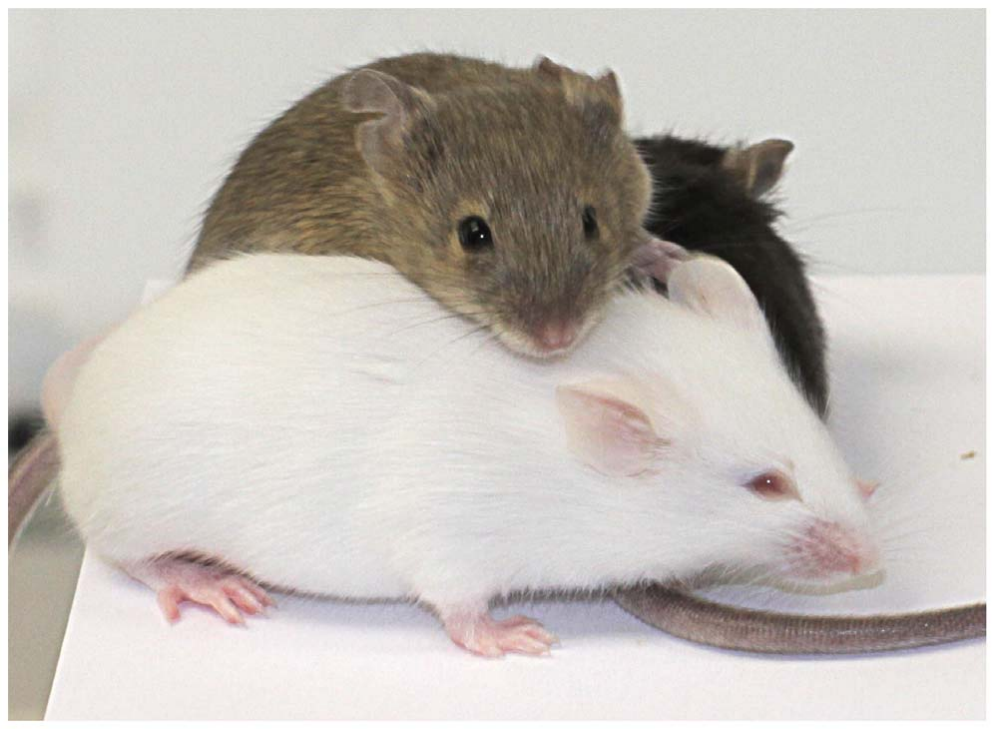
F2 offspring coat colors. Intercrosses between C57BL/6NTac-*^Atm1.1Arte^* and C57BL/6NTac-*Tyr^tm1Arte^* display all different possible coat colors (black, agouti, albino). The double mutant C57BL/6NTac-*^Atm1.1Arte^Tyr^tm1Arte^* (front, termed Albino A++) is phenotypically albino.

Intercrossing of both strains to homozygosity resulted in the phenotypically albino double mutant C57BL/6NTac-*A^tm1.1Arte^Tyr^tm1Arte^* (referred as Albino A^++^) (Fig. 3).

The C57BL/6NTac substrain specificity and the introduced tyrosinase base pair mutation were confirmed by Single Nucleotide Polymorphism (SNP) analysis (Fig. S1).

**Figure 4:**
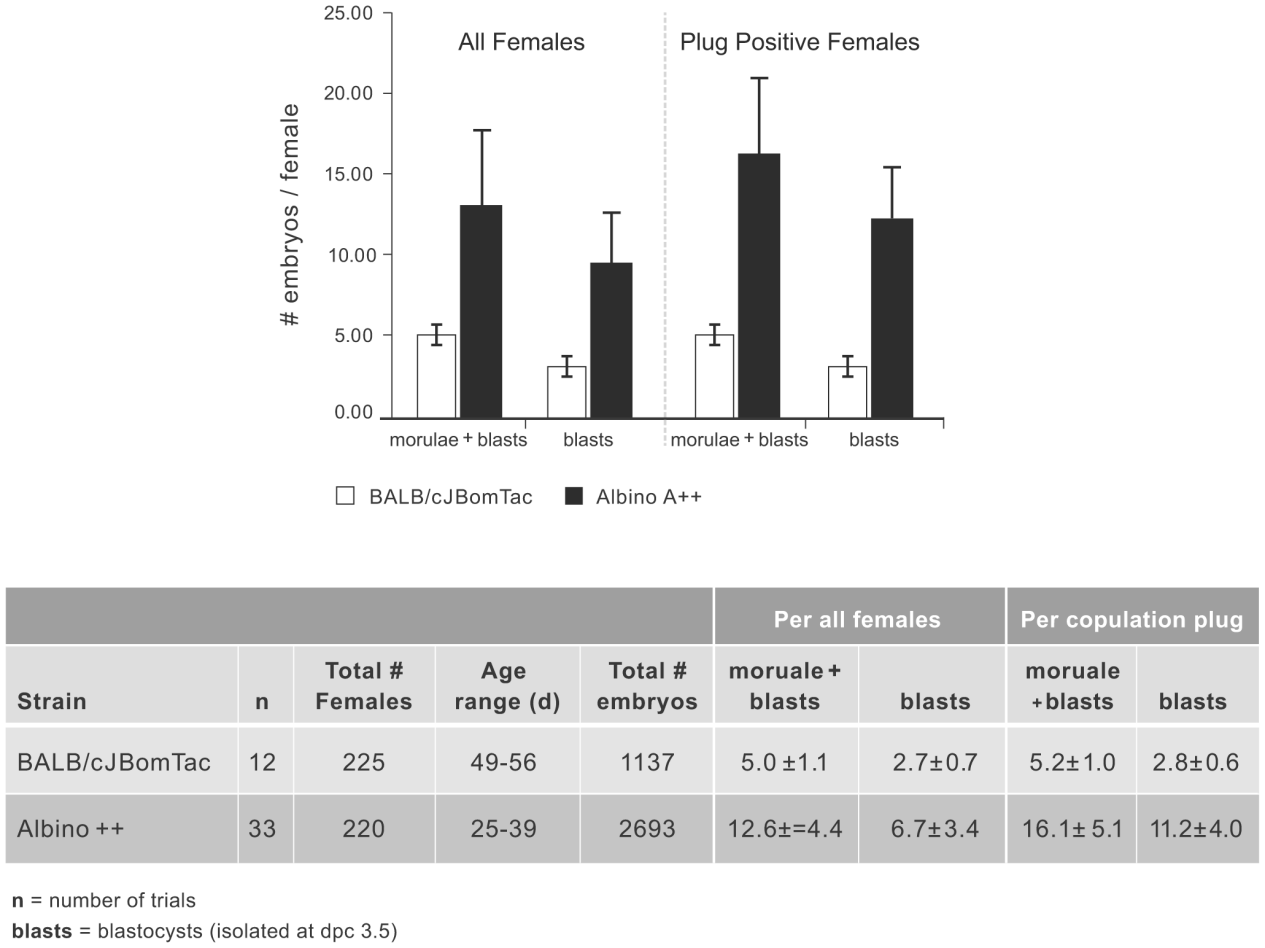
Embryo yield upon superovulation of Albino A++ in comparison to BALB/cJBomTac. The graph displays average numbers of embryos per superovulated female (left panel) or plug positive female (right panel) harvested from strains BALB/cJBomTac (white columns) or Albino A++ (black columns). Details including total number of experiments (n) and females assayed (total # females) are outlined in the attached table. The average number of embryos harvested from Albino A++ females is in all cases significantly higher compared to BALB/cJBomTac (p<0.0001).

We compared embryo production upon superovulation of Albino A++ to BALB/cJBomTac females, which is the strain routinely used at Taconic for microinjection of B6NTac ES cells (Fig. 4). Blastocysts and morulae were included in the embryo count, as morulae can be used for injection upon in vitro development to blastocysts [7]. On average, 5.0±1.1 embryos were isolated per superovulated BALB/cJBomTac female at dpc 3.5. In contrast, the superovulation of double mutant Albino++ females yielded 12.6±4.4 embryos per female, which is significantly higher and consistent with our data on superovulation of C57BL/6NTac (unpublished results). The difference is even more pronounced if the embryo yield is determined only from copulation plug positive females (5.2±1.0 vs. 16.1±5.1). In addition, the ratio of immediately suitable blastocysts for microinjection is higher in Albino A++ (70%) compared to BALB/cJBomTac (53%, p<0.0001).

Finally, we tested the suitability of freshly isolated Albino A++ blastocysts as embryo host for the production of chimeras. Microinjection of 21 different gene targeted C57BL/6NTac ES cell clones resulted in 156 chimeras (77% of all live born pups evaluated). 91% of those were identified as males, which is indicative of a strong contribution of the introduced male ES cells to produce sex conversion of female host embryos. 57% of all males were judged by coat color as a minimum of 50% chimeric (Table 1 and Data S1). Altogether, these results are superior or equally good to previously published results on chimera production with targeted C57BL/6N and C57BL/6J ES cells [3], [6], [11], [12], [18], [19]. Interestingly, chimeric coat colors obtained by injection of B6 ES cells into Albino A++ hosts differ from those obtained by injection into BALB/c hosts. Even strong B6/Albino A++ chimeras appear to display more agouti/brown coat than the commonly achieved agouti/black appearance of B6/BALB/c chimeras. We speculate that chimeric coat color differences are influenced by allelic differences of another enzyme of the tyrosinase family, the tyrosinase-related protein 1 (*Tyrp1*) locus. BALB/c mice encode the mutated *Tyrp1^b^* allele while C57BL/6NTac has the wild type *Tyrp1* allele.

**Table 1:** Generation of chimeras and germline transmission of C57BL/6NTac targeted ES clones injected into Albino A++ and Albino host embryos.

	Albino A++ (%)		Albino (%)	
**# C57BL/6Ntac ES Clones injected**	21		3	
**# pups born**	210		20	
**# chimeras born (% of live born)**	156	74%	19	95%
**# male chimeras weaned (% of chimeras born)**	142	91%	12	63%
**# >50% chimeric males (% of chimeras born)**	89	57%	9	47%
**# clones test mated**	6		2	
**# chimeras tested**	17		5	
**# chimeras sterile**	3		0	
**# GLT** * **chimeras (% of chimeras mated)**	14	82%	5	100%
**# GLT clones (% of clones mated)**	6	100%	2	100%

* GLT indicates germline Transmission.

Alternatively, the observed coat color differences may indicate that C57BL/6NTac ES cell participation to embryo development is in fact inferior in the co-isogenic Albino A++ host environment as compared to BALB/c. However, the ultimate proof of the suitability of the Albino and Albino A++ strains is the ability of B6 chimeras to transmit the introduced B6 ES cell genome to the G1 generation. 22 male chimeras (minimum 50% coat color chimerism) generated from 8 independent gene targeted B6 ES cell clones were mated for this purpose to C57BL/6NTac mice (Table 1, Table S1 and Data S2). Germline transmission was obtained from 14 (82%) male chimeras derived from Albino A++ blasts, judged by coat color of G1 offspring. The remaining 3 chimeras did not sire litter within the breeding period (min. 10 weeks). 9 chimeras generated on the Albino A++ background contributed 100% of the ES cell genome to their offspring. Similar results were obtained with the single Albino mutant. The fact that such high germline transmission rates are achieved with chimeras produced with ES cell clones randomly chosen from unrelated targeted alleles without further quality control such as karyotype analysis, demonstrates the high suitability of B6 chimera production using the Albino and Albino A++ from C57BL/6 genetic background.

Taken together, we have generated a C57BL/6NTac homozygous double mutant mouse line with a restored agouti locus and deficiency in tyrosinase function. This mouse model is an ideal host for the generation of targeted mutant mouse models from available C57BL/6 ES cell resources [20], but not only confined to this genetic background (Table S2). We have demonstrated that the C57BL/6NTac albino A++ enables the efficient production of chimeras, the visualisation of highly chimeric animals, the identification of B6NTac ES cell derived G1 offspring by coat color, and that the derivation of such offspring is possible on a pure C57BL/6NTac background at the sub-strain level, thereby eliminating the need to backcross congenic lines to the C57BL/6 reference strain. As neither superovulation nor ES cell transfer protocols had been optimized for this study, it is likely that efficiencies can be further improved. Independently of applications for transgenic research, Albino A++ mice may also serve as a better suited model for imaging on a pure C57BL/6NTac background, such as xenograft applications, as white fur does not interfere with emission of light.

## Materials and Methods

### Ethics statement

The animal study protocol was approved according to the German Animal Welfare Act (§ 8 (1) TierSchG) by the local authority (animal study protocol AZ 9.93.2.10.34.07.190). BALB/cJBomTac mice were obtained from TaconicEurope. NMRI females were used as pseudopregnant recipients. Animals were maintained at TaconicArtemis GmbH in microisolator cages (Tecniplast Sealsave). Feed and water were available *ad libitum*. Light cycles were on a 12∶12 h light:dark cycle with the light phasing starting at 06:00 h. Temperature and relative humidity were maintained between 21 and 23°C and 45 and 65%.

### Construction of the nonagouti targeting vector

The targeting vector was based on two genomic fragments from the nonagouti (*a*) gene encompassing exon 1 and flanking sequences. The homology arms with a total length of 12.7-kb were positioned directly 5′ and 3′ of the 14.7 kb retrotransposon embedded within intron 1 (NCBI gene ID 5018) to allow for the genomic replacement of the retrotransposon sequence by an FRT-flanked Neomycin/Puromycin resistance cassette. A Thymidine Kinase (Tk) cassette was inserted at the 3′ end of the genomic fragment (not shown).

### Construction of the tyrosinase targeting vector

The targeting vector was based on a 10.3-kb genomic fragment from the tyrosinase (*Tyr*) gene encompassing exon 1 and flanking sequences (NCBI gene ID: 22173). This fragment, obtained from the C57Bl/6J RP23 BAC library, was modified by inserting the point mutation C103S in exon 1. An F3-flanked Puromycin resistance gene was inserting into intron 1 and a Thymidine Kinase (Tk) cassette was inserted at the 3′ end of the genomic fragment (not shown).

### Embryonic stem (ES) cell culture and generation of nonagouti and tyrosinase targeted ES cells

C57BL/6NTac ES cells (B6-3) [21] were grown on a mitotically inactivated feeder layer comprised of mouse embryonic fibroblasts (MEF) in DMEM High Glucose medium containing 20% FBS (PAN Biotech GmbH) and 1200 u/mL Leukemia Inhibitory Factor (Miltenyi). 1×10^7^ cells and 30 µg of linearized DNA targeting vector were electroporated (Biorad Gene Pulser) at 240 V and 500 µF. Positive selection of tyrosinase targeted ES cells with Puromycin (1 µg/mL) started on day 2; for nonagouti locus targeted ES cells a double positive selection with Puromycin (1 µg/mL) and G418 (250 µg/mL) was applied. Counterselection with Gancyclovir (2 µM) started on day 5 after electroporation for both transfections. Resistant ES cell colonies with a distinct morphology were isolated on day 8 after transfection and expanded in 96well plates. Correctly recombined ES cell clones were identified by Southern Blotting according to standard procedures using external and internal probes and frozen in liquid nitrogen. The external probe 1 was amplified with oligos CTGGGAAAGTGCACTCCTTCTGG and GTAATGACAATGAAATGGCCAC, oligos GGATCAGGTCCTCCCTCTGCACAG and CAGACACATCTTTAGAGGCAAC were used to generate external probe 2.

### In-vitro deletion of the selection marker in tyrosinase targeted ES cells

Upon fluorescence in situ hybridization for count of chromosomes 8, 11, X and Y (Chrombios GmbH, Raubling, Germany) the selection marker cassette of tyrosinase locus targeted ES cell clones was deleted in vitro. 4×10^6^ cells were used for nucleofection with 20 µg of circular pCAGGS-Flpe-pA [22] according to manufacturer's protocol (Amaxa Nucleofector™). 2 days after transfection, cells were replated at low density and grown until individual clones were visible. Clones were isolated on day 8 and duplicates from each clone were tested for sensitivity against the selection marker. Sensitive clones were expanded, verified by PCR and frozen in liquid nitrogen.

### Generation of tyrosinase and nonagouti mutant mice

Albino A++ females were superovulated between 3.5 and 5.5 weeks of age, BALB/cJBomTac were superovulated between 7 and 8 weeks of age. 5 I.U. of the two gonadotropins PMSG (pregnant mare's serum gonadotropin, ReboPharm) and hCG (human chorionic gonadotropin, ReboPharm) hormones were administered by i.p. injection. HCG was administered 47 h after PMSG. Blastocysts were isolated from the uterus at dpc 3.5 upon mating by flushing the uteri with KSOM medium (Sigma). For microinjection, blastocysts were placed in a drop of DMEM with 15% FCS under mineral oil. A flat tip, piezo actuated microinjection-pipette with an internal diameter of 12–15 micrometer was used to inject 10–12 targeted B6-3 ES cells into each blastocyst. After recovery, 8 injected blastocysts were transferred to each uterine horn of 2.5 days post coitum, pseudopregnant NMRI females. Chimerism was measured by coat color contribution of ES cells to the BALB/c host. Chimeras generated from injection of restored dominant agouti targeted ES cells (ESC) were phenotypically agouti on white. For germline transmission C57BL/6NTac-*Tyr^tm1Arte^* chimeric males were bred to strain C57BL/6NTac females, whereas C57BL/6NTac-*A^tm1Arte^* chimeric males were bred to C57BL/6NTac-Tg(CAG-Flpe)2Arte females to simultaneously delete the introduced antibiotic selection marker. Germline transmission in G1 offspring was verified by PCR. Both strains are made available by Taconic Europe and Taconic US.

### PCR genotyping of ESC clones and mice

Genomic DNA was extracted from 1- to 2-mm-long tail tips using the NucleoSpin Tissue kit (Macherey-Nagel). Genomic DNA (2 µl) was analyzed by PCR in a final volume of 50 µl in the presence of 2.0 mM MgCl2, 200 µM dNTPs, 100 nM of each primer, and 2 U of Taq DNA polymerase (Invitrogen). Following a denaturing step at 95°C for 5 minutes, 35 cycles of PCR were performed, each consisting of a denaturing step at 95°C for 30 seconds, followed by an annealing phase at 60°C for 30 seconds and an elongation step at 72°C for 1 minute. PCR was finished by a 10-minute extension step at 72°C. The PCR amplicons were analyzed by using a Caliper LabChip GX device (Caliper Life Sciences Inc. MA, USA).

### Detection of restored and wildtype agouti alleles

Primers a (GGAATTGAGAGAGGCTGTTCC) and b (ATGACTGAACTTCTGGCTCTCC) detect the presence of the heterozygous/homozygous *A^tm1.1^* allele (387 bp) in Albino A++ and *A* allele in BALB/c (290 bp).

### Detection of the nonagouti allele

Primers c (GCCAGTAATTTTTCATTCTTCAGC) and b (ATGACTGAACTTCTGGCTCTCC) detect the presence of the nonagouti *a* allele (280 bp) in C57BL/6NTac.

### Detection of the modified tyrosinase allele

Primers f (GCATTGTTGGTAAATAGCAAAGG) and g (AGAAGGCTAATTTTTCTCCATCC) detect the presence of the constitutive tyrosinase knock-in (*Tyr^tm1^*) allele (242 bp) and the tyrosinase wildtype (W) allele (151 bp).

### Verification of the introduced C103S point mutation

Primers d CAGCTTTCAGGCAGAGGTTC and e CCATACAAAGAGGTCGTAGATG were applied detecting the presence of heterozygous/homozygous (*Tyr^tm1^*) and wildtype alleles (393 bp) followed by sequencing.

### SNP analysis

Genomic DNA was prepared from tail biopsies using a Qiagen Genomic DNA isolation kit following manufacturer's recommendations. SNP analysis was performed using Illumina's Golden Gate platform. There are 22 SNPs that are polymorphic between C57BL/6NTac and C57BL/6J and one specific SNP showing the introduced point mutation in the Tyrosinase gene. (Fig. S1)

### Statistical analysis

The statistical significance of differences in superovulation in different mouse strains and ages was calculated by ANOVA and regression analysis, respectively using the statistics software package, R 3.00 (www.r-project.org).

## Supporting Information

Figure S1 **SNP Analysis.**
(PDF)

Table S1 **Germline Transmission Performance.**
(DOCX)

Table S2 **Coat color detection and genotype schemes.**
(PDF)

Data S1 **Chimera production.**
(XLSX)

Data S2 **Chimera Matings.**
(XLSX)

## References

[1] Collins FS, Rossant J, Wurst W (2007) A mouse for all reasons. Cell 128: 9–13. 17218247 10.1016/j.cell.2006.12.018

[2] Ledermann B, Burki K (1991) Establishment of a germ-line competent C57BL/6 embryonic stem cell line. Experimental Cell Research 197: 254–258. 1959560 10.1016/0014-4827(91)90430-3

[3] Schuster-Gossler K, Lee AW, Lerner CP, Parker HJ, Dyer VW, et al. (2001) Use of coisogenic host blastocysts for efficient establishment of germline chimeras with C57BL/6J ES cell lines. Biotechniques 31: : 1022–1024, 1026. 10.2144/01315st01 11730008

[4] Cheng J, Dutra A, Takesono A, Garrett-Beal L, Schwartzberg PL (2004) Improved generation of C57BL/6J mouse embryonic stem cells in a defined serum-free media. Genesis 39: 100–104. 15170695 10.1002/gene.20031

[5] Kontgen F, Suss G, Stewart C, Steinmetz M, Bluethmann H (1993) Targeted disruption of the MHC class II Aa gene in C57BL/6 mice. International Immunology 5: 957–964. 8398989 10.1093/intimm/5.8.957

[6] Auerbach W, Dunmore JH, Fairchild-Huntress V, Fang Q, Auerbach AB, et al. (2000) Establishment and chimera analysis of 129/SvEv- and C57BL/6-derived mouse embryonic stem cell lines. Biotechniques 29: : 1024–1028, 1030, 1032. 10.2144/00295st04 11084865

[7] Lemckert FA, Sedgwick JD, Korner H (1997) Gene targeting in C57BL/6 ES cells. Successful germ line transmission using recipient BALB/c blastocysts developmentally matured in vitro. Nucleic Acids Research (Online) 25: 917–918. 10.1093/nar/25.4.917 PMC146508 9016649

[8] Nagy A, Gertsenstein M, Vintersten K, Behringer R (2003) Manipulating the mouse embryo: a laboratory manual. Cold Spring Harbor, NY: Cold Spring Harbor Laboratory Press. VIII, 764 S. p.

[9] Ohta H, Sakaide Y, Wakayama T (2009) Age- and substrain-dependent sperm abnormalities in BALB/c mice and functional assessment of abnormal sperm by ICSI. Hum Reprod 24: 775–781. 19098292 10.1093/humrep/den456

[10] Pacholczyk G, Suhag R, Mazurek M, Dederscheck SM, Koni PA (2008) Generation of C57BL/6 knockout mice using C3H x BALB/c blastocysts. Biotechniques 44: 413–416. 18361795 10.2144/000112706

[11] Fielder TJ, Yi CS, Masumi J, Waymire KG, Chen HW, et al.. (2012) Comparison of male chimeric mice generated from microinjection of JM8.N4 embryonic stem cells into C57BL/6J and C57BL/6NTac blastocysts. Transgenic Res. 10.1007/s11248-012-9605-3 PMC3445772 22422470

[12] Pettitt SJ, Liang Q, Rairdan XY, Moran JL, Prosser HM, et al (2009) Agouti C57BL/6N embryonic stem cells for mouse genetic resources. Nat Methods 6: 493–495. 19525957 10.1038/nmeth.1342 PMC3555078

[13] Ryder E, Wong K, Gleeson D, Keane TM, Sethi D, et al (2013) Genomic analysis of a novel spontaneous albino C57BL/6N mouse strain. Genesis 51: 523–528. 23620107 10.1002/dvg.22398 PMC3799019

[14] Bultman SJ, Michaud EJ, Woychik RP (1992) Molecular characterization of the mouse agouti locus. Cell 71: 1195–1204. 1473152 10.1016/s0092-8674(05)80067-4

[15] Bultman SJ, Klebig ML, Michaud EJ, Sweet HO, Davisson MT, et al (1994) Molecular analysis of reverse mutations from nonagouti (a) to black-and-tan (a(t)) and white-bellied agouti (Aw) reveals alternative forms of agouti transcripts. Genes Dev 8: 481–490. 8125260 10.1101/gad.8.4.481

[16] Kwon BS, Haq AK, Wakulchik M, Kestler D, Barton DE, et al (1989) Isolation, chromosomal mapping, and expression of the mouse tyrosinase gene. J Invest Dermatol 93: 589–594. 2507645 10.1111/1523-1747.ep12319693

[17] Yokoyama T, Silversides DW, Waymire KG, Kwon BS, Takeuchi T, et al (1990) Conserved cysteine to serine mutation in tyrosinase is responsible for the classical albino mutation in laboratory mice. Nucleic Acids Res 18: 7293–7298. 2124349 10.1093/nar/18.24.7293 PMC332865

[18] Gertsenstein M, Nutter LM, Reid T, Pereira M, Stanford WL, et al (2010) Efficient generation of germ line transmitting chimeras from C57BL/6N ES cells by aggregation with outbred host embryos. PLoS ONE 5: e11260. 20582321 10.1371/journal.pone.0011260 PMC2889837

[19] Schoonjans L, Kreemers V, Danloy S, Moreadith RW, Laroche Y, et al (2003) Improved generation of germline-competent embryonic stem cell lines from inbred mouse strains. Stem Cells 21: 90–97. 12529555 10.1634/stemcells.21-1-90

[20] Skarnes WC, Rosen B, West AP, Koutsourakis M, Bushell W, et al (2011) A conditional knockout resource for the genome-wide study of mouse gene function. Nature 474: 337–342. 21677750 10.1038/nature10163 PMC3572410

[21] Kern H, Zevnik B (2009) ES Cell Line Establishment. In: Wurst W, Kühn R, editors. Gene Knockout Protocols: Humana Press. pp. 187–204.

[22] Schaft J, Ashery-Padan R, van der Hoeven F, Gruss P, Stewart AF (2001) Efficient FLP recombination in mouse ES cells and oocytes. Genesis 31: 6–10. 11668672 10.1002/gene.1076

