# Supplementary material for: C57BL/6N Albino/Agouti Mutant Mice as Embryo Donors for Efficient Germline Transmission of C57BL/6 ES Cells
Source: PLoS One. 2014 Mar 5;9(3):e90570. doi: 10.1371/journal.pone.0090570 (PMC3944090; doi:10.1371/journal.pone.0090570)
Supplement: Figure S1 — SNP Analysis. (PDF) [file pone.0090570.s001.pdf]

Figure S1 SNP Analysis

SNP	Chr	Position	C57BL/6NTac	albino A++	C57BL6/J
Mus-Ptprc	1	52665170	C	C	C
rs13476148	1	142911628	A	A	C
CEL-2_23847726	2	23847726	C	C	A
rs13476554	2	67180899	T	T	A
rs13477019	3	23589162	A	A	T
Mus-Tyrp1B	4	80481305	G	G	G
rs13477622	4	28491198	G	G	A
rs3662161	5	114905705	G	G	A
rs13478783	6	60682681	G	G	A
rs13478995	6	117862153	C	C	G
Mus-TyrC	7	94641553	G	**C**	G
rs13479522	7	116540646	G	G	A
rs13480100	9	21200544	G	G	A
rs13480122	9	31136193	G	G	A
rs13480619	10	57805922	G	G	A
rs29359333	10	57796761	T	T	C
rs13480759	10	109059096	A	A	G
rs13481014	11	47757117	G	G	A
rs13481573	12	82237479	A	A	G
rs13481634	12	101558810	C	C	A
rs13481734	13	26416832	G	G	A
CEL-14_116404928	14	116404928	A	A	G
rs4165065	16	17188907	G	G	A
rs13483055	17	58655424	G	G	A
rs13483237	18	19671420	G	G	C

SNP confirmation of the C57BL/6NTac substrain identity of Albino A++ (blue), compared to C57BL/6J (red). The gene-targeted *Tyr* nucleotide exchange from G to C resulting in a C103S amino acid substitution in Albino A++ is highlighted.
