# Supplementary material for: C57BL/6N Albino/Agouti Mutant Mice as Embryo Donors for Efficient Germline Transmission of C57BL/6 ES Cells
Source: PLoS One. 2014 Mar 5;9(3):e90570. doi: 10.1371/journal.pone.0090570 (PMC3944090; doi:10.1371/journal.pone.0090570)
Supplement: Table S1 — Germline Transmission Performance. (DOCX) [file pone.0090570.s002.docx]

| **Host** | **ES cell line** | **Clone** | **Fertile male chimeras** | **# GLT/**  **fertile chimera** | **# 100% GLT/**  **GLT chimera** | **# GLT pups/**  **total # pups** |
| --- | --- | --- | --- | --- | --- | --- |
| Albino A++ | C57BL/6NTac | A | 1/2 (50%) | 1/1 | 1/1 | 33/33 (100%) |
|  | C57BL/6NTac | B | 2/3 (67%) | 2/2 | 2/2 | 33/33 (100%) |
|  | C57BL/6NTac | C | 2/3 (67%) | 2/2 | 2/2 | 26/26 (100%) |
|  | C57BL/6NTac | D | 4/4 (100%) | 4/4 | 1/4 | 10/50 (25%) |
|  | C57BL/6NTac | E | 3/3 (100%) | 3/3 | 3/3 | 42/42 (100%) |
|  | C57BL/6NTac | F | 2/2 (100%) | 2/2 | 0/2 | 10/29 (34%) |
|  |  |  |  |  |  |  |
| Albino | C57BL/6NTac | G | 2/2 (100%) | 2/2 | 1/2 | 67/70 (96%) |
|  | C57BL/6NTac | H | 3/3 (100%) | 3/3 | 2/3 | 36/67 (54%) |

**Supplementary Table S1** | Germline Transmission performance per individual targeted ES clone and germline transmitting chimera; GLT = GermLine Transmission.
