# Supplementary material for: C57BL/6N Albino/Agouti Mutant Mice as Embryo Donors for Efficient Germline Transmission of C57BL/6 ES Cells
Source: PLoS One. 2014 Mar 5;9(3):e90570. doi: 10.1371/journal.pone.0090570 (PMC3944090; doi:10.1371/journal.pone.0090570)
Supplement: Table S2 — Coat color detection and genotype schemes. (PDF) [file pone.0090570.s003.pdf]

Supplementary Table S2 | Coat color detection and genotype schemes of different Albino host <-> ES cell and chimera <-> C57BL/6 (a) or Albino host strain (b) mating partner combinations. Only (non-)agouti and tyrosinase coat color alleles are considered.

I) Albino A++ (C57BL/6NTac-$A^{tm1.1Arte}Tyr^{tm1Arte}$), 100% recognition of GLT transmission by coat color with combinations 1a, 1b and 2b, note transmission of the $A^{tm1.1}$ and Tyr^{tm1} allele with 1b and 2b.

II) Albino (C57BL/6NTac-$A^{tm1.1Arte}$), 100% recognition of GLT transmission by coat color only with combination 1b, note transmission of the Tyr^{tm1} allele.

I) Albino A++ host Strain: C57BL/6NTac-$A^{tm1.1Arte}Tyr^{tm1}$ (allele configuration: $A^{tm1.1}/A^{tm1.1}$, Tyr^{tm1}/Tyr^{tm1})

	ES cell	Chimera Coat Color	Mating Partner	Offspring Non-Germline	Offspring Germline
1a	B6.3-6, JM8, C57BL/6NTac (a/a, Tyr/Tyr)	C57BL/6NTac ($A^{tm1.1}/A^{tm1.1}$, Tyr^{tm1}/Tyr^{tm1}) + C57BL/6NTac (a/a, Tyr/Tyr)	C57BL/6NTac (a/a, Tyr/Tyr)	C57BL/6NTac ($A^{tm1.1}$/a, Tyr^{tm1}/Tyr)	C57BL/6NTac (a/a, Tyr/Tyr)
1b	B6.3-6, JM8, C57BL/6NTac (a/a, Tyr/Tyr)	C57BL/6NTac ($A^{tm1.1}/A^{tm1.1}$, Tyr^{tm1}/Tyr^{tm1}) + C57BL/6NTac (a/a, Tyr/Tyr)	C57BL/6NTac ($A^{tm1.1}/A^{tm1.1}$, Tyr^{tm1}/Tyr^{tm1})	C57BL/6NTac ($A^{tm1.1}/A^{tm1.1}$, Tyr^{tm1}/Tyr^{tm1})	C57BL/6NTac (a/$A^{tm1.1}$/, Tyr/Tyr^{tm1})
2a	JM8A.3 (C57BL/6NTac) (A^{tm1Brd}/a, Tyr/Tyr)	C57BL/6NTac ($A^{tm1.1}/A^{tm1.1}$, Tyr^{tm1}/Tyr^{tm1}) + C57BL/6NTac (A^{tm1Brd}/a, Tyr/Tyr)	C57BL/6NTac (a/a, Tyr/Tyr)	C57BL/6NTac ($A^{tm1.1}$/a, Tyr^{tm1}/Tyr)	C57BL/6NTac (a/a, Tyr/Tyr)<br>C57BL/6NTac (A^{tm1Brd}/a, Tyr/Tyr)
2b	JM8A.3 (C57BL/6NTac) (A^{tm1Brd}/a, Tyr/Tyr)	C57BL/6NTac ($A^{tm1.1}/A^{tm1.1}$, Tyr^{tm1}/Tyr^{tm1}) + C57BL/6NTac (A^{tm1Brd}/a, Tyr/Tyr)	C57BL/6NTac ($A^{tm1.1}/A^{tm1.1}$, Tyr^{tm1}/Tyr^{tm1})	C57BL/6NTac ($A^{tm1.1}/A^{tm1.1}$, Tyr^{tm1}/Tyr^{tm1})	C57BL/6NTac<br>50% (a/$A^{tm1.1}$, Tyr/Tyr^{tm1})<br>50% ($A^{tm1Brd}/A^{tm1.1}$, Tyr/Tyr^{tm1})

II) Albino host Strain: C57BL/6NTac-$Tyr^{tm1.1Arte}$ (allele configuration: a/a, Tyr^{tm1}/Tyr^{tm1})

	ES cell	Chimera Coat Color	Mating Partner	Offspring Non-Germline	Offspring Germline
1a	B6.3-6, JM8, C57BL/6NTac (a/a, Tyr/Tyr)	C57BL/6NTac (a/a, Tyr^{tm1}/Tyr^{tm1}) + C57BL/6NTac (a/a, Tyr/Tyr)	C57BL/6NTac (a/a, Tyr/Tyr)	C57BL/6NTac (a/a, Tyr^{tm1}/Tyr)	C57BL/6NTac (a/a, Tyr/Tyr)
1b	B6.3-6, JM8, C57BL/6NTac (a/a, Tyr/Tyr)	C57BL/6NTac (a/a, Tyr^{tm1}/Tyr^{tm1}) + C57BL/6NTac (a/a, Tyr/Tyr)	C57BL/6NTac (a/a, Tyr^{tm1}/Tyr^{tm1})	C57BL/6NTac (a/a, Tyr^{tm1}/Tyr^{tm1})	C57BL/6NTac (a/a, Tyr/Tyr^{tm1})
2a	JM8A.3 (C57BL/6NTac) (A^{tm1Brd}/a, Tyr/Tyr)	C57BL/6NTac (a/a, Tyr^{tm1}/Tyr^{tm1}) + C57BL/6NTac (A^{tm1Brd}/a, Tyr/Tyr)	C57BL/6NTac (a/a, Tyr/Tyr)	C57BL/6NTac (a/a, Tyr^{tm1}/Tyr)	C57BL/6NTac (a/a, Tyr/Tyr)<br>C57BL/6NTac (A^{tm1Brd}/a, Tyr/Tyr)
2b	JM8A.3 (C57BL/6NTac) (A^{tm1Brd}/a, Tyr/Tyr)	C57BL/6NTac (a/a, Tyr^{tm1}/Tyr^{tm1}) + C57BL/6NTac (A^{tm1Brd}/a, Tyr/Tyr)	C57BL/6NTac (a/a, Tyr^{tm1}/Tyr^{tm1})	C57BL/6NTac (a/a, Tyr^{tm1}/Tyr^{tm1})	C57BL/6NTac (a/a, Tyr/Tyr^{tm1})<br>C57BL/6NTac (A^{tm1Brd}/a, Tyr/Tyr^{tm1})
